# Application of artificial intelligence in predicting lymph node metastasis in breast cancer

**DOI:** 10.3389/fradi.2023.928639

**Published:** 2023-02-20

**Authors:** Gabrielle O. Windsor, Harrison Bai, Ana P. Lourenco, Zhicheng Jiao

**Affiliations:** ^1^ Department of Diagnostic Imaging, Brown University, Providence, RI, United States; ^2^ Department of Radiology and Radiological Sciences, Johns Hopkins University, Baltimore, MD, United States

**Keywords:** artificial intelligence, deep learning, radiology, axillary lymph node metastasis, breast cancer

## Abstract

Breast cancer is a leading cause of death for women globally. A characteristic of breast cancer includes its ability to metastasize to distant regions of the body, and the disease achieves this through first spreading to the axillary lymph nodes. Traditional diagnosis of axillary lymph node metastasis includes an invasive technique that leads to potential clinical complications for breast cancer patients. The rise of artificial intelligence in the medical imaging field has led to the creation of innovative deep learning models that can predict the metastatic status of axillary lymph nodes noninvasively, which would result in no unnecessary biopsies and dissections for patients. In this review, we discuss the success of various deep learning artificial intelligence models across multiple imaging modalities in their performance of predicting axillary lymph node metastasis.

## Introduction

Breast cancer remains a prevalent disease both globally and within the U.S., with approximately 1 in 8 U.S. women estimated to develop invasive breast cancer during their lifetime (1). One of the hallmarks of cancer includes its propensity to metastasize to distant regions of the body, leading to a worse prognosis for the patient. When breast cancer metastasizes, it generally spreads first to the axillary lymph nodes, due to their proximity in the underarm region. Therefore, axillary lymph nodes aid in determining breast cancer stage through the TNM Classification of Malignant Tumors as well as provide a helpful guide for treatment (1, 2). Additionally, the extent of axillary lymph node metastasis remains the most reliable predictor of prognosis for the patient (3–5). Axillary lymph nodes therefore are clinically important in the treatment of breast cancer patients.

The current gold standard of determining the involvement of axillary lymph nodes in a patient with breast cancer includes a pathological examination of aspiration cytology, a sentinel lymph node biopsy to detect metastasis from the primary tumor, and, in some cases, an axillary lymph node dissection. Both sentinel lymph node biopsy and axillary dissection are invasive procedures conducted under general anesthesia, with associated risks and clinical complications, including infection, edema, changes in sensation, chronic pain, and axillary web syndrome (6, 7). Additionally, some patients undergo unnecessary axillary lymph node dissection. According to some studies, an estimated 43%–65% of patients with sentinel lymph node metastasis also undergoing axillary lymph node dissection had no additional metastatic lymph nodes (6). Multiple studies have shown that, for many patients, sentinel lymph node biopsy results in similar patient outcomes (Z11 and NSABP B-32 studies) while minimizing patient morbidity, thereby leading to fewer axillary lymph node dissections. Creating and standardizing a non-invasive method of evaluating both sentinel and axillary lymph node metastatic status would be beneficial for breast cancer patients.

Artificial Intelligence (AI), especially deep learning algorithms that utilize convolutional neural networks (CNN), has grown in popularity within the medical community for revolutionizing image-based disease diagnosis (8–12). Imaging modalities used to visualize axillary lymph nodes in breast cancer patients on their own have a wide range of sensitivity and specificity, and some are dependent on operator performance (13). For example, Ultrasound and PET/CT demonstrate a sensitivity of 33%–86.2% and 20%–80%, respectively, and a specificity of 40.5%–96.2% and 88.6%–97%, respectively (13). AI algorithms have the potential to enhance the diagnostic capability of such imaging modalities and shift clinical standards to a non-invasive, preoperative staging of lymph node involvement. In this review, we examine recent studies demonstrating the success of deep learning (DL) AI systems in the detection of axillary lymph node metastasis in breast cancer patients.

## The role of traditional machine learning and deep learning in mammography

In recent years, the combination of screening programs and increased incidence of breast cancer together has resulted in an enlarged workload for radiologists. Computer-aided detection/diagnosis (CAD) systems, mostly in form of traditional machine learning (ML) AI methods, were created in part to assist radiologists with such high volumes of breast imaging. They have been used to detect lesions of interest in breast imaging as well as to differentiate between benign and malignant breast tissue (14, 15). Following traditional ML strategies, CAD systems rely on pre-defined/hand-crafted features inputted into the algorithm. These systems will learn from the tasks that they complete and subsequently improve their accuracy. Additionally, CAD systems are versatile in their ability to function with a wide range of imaging modalities (i.e.,: ultrasound, mammography, computed tomography, magnetic resonance imaging, etc.). Initial evidence suggested that CAD systems helped to improve accuracy in both detection and diagnosis of malignant breast lesions, however, largescale retrospective studies now demonstrate that CAD systems do not improve diagnostic accuracy, instead offering no benefit or, in some cases, reducing radiologist accuracy (14–16). DL-based CAD systems, in contrast, vastly outperform traditional ML-based ones on radiology tasks (16).

DL is a subset of ML in which raw data is given to a program and the algorithm itself is responsible for determining and defining the features, without any human interference. Its ability to learn from the given data (in this case, medical imaging) necessitates a large amount of data for the system to have higher levels of accuracy (16). CNNs are a specific type of DL algorithm that determine features in images and are popular in analyzing breast cancer images. To assess the performance of the DL algorithms discussed throughout this review, we will examine the Area Under the Curve of Receiver Characteristic Operator (AUROC) (14).

## Deep learning in ultrasound, computed tomography, and magnetic resonance imaging

Recent studies investigating the prediction of axillary lymph node metastasis on ultrasound through DL systems have shown great success. Ultrasound is the primary imaging tool when examining axillary lymph nodes, due in part to the imaging modality offering a non-invasive, low cost, non-ionizing radiation method of visualizing a patient's lymph nodes in real time (14, 17). Additionally, ultrasound offers the option of immediate image guided intervention. Despite the many advantages associated with utilizing ultrasound to visualize axillary lymph nodes preoperatively, the medical community has yet to reach an official consensus on creating criteria that classifies axillary lymph nodes as either benign or malignant (17). A recent study by Tahmasebi et al. examines an AI system through Google Cloud AutoML Vision that classifies preoperative axillary lymph nodes of breast cancer patients as either benign or malignant and compares the results against blind readings from three experienced radiologists. The study reports the AI performed comparably to the trained radiologists, with less sensitivity (AI was 74.0% ± 0.14% compared to radiologist at 89.9% ± 0.06%) and more specificity (AI was 64.4% ± 0.11% compared to radiologist at 50.1% ± 0.20%) in external validation—though the differences between the AI and radiologist group were not statistically significant—and concludes that utilizing a combination of AI system and radiologist in practice may optimize results. Additionally, the study was retrospective in nature, and resulted in the limitation of ultrasound images lacking standardization in image axis, plane, and view (17). Another study by Sun et al. investigates a custom CNN with a total of 12 convolutional layers in the model for the prediction of axillary lymph node metastasis using ultrasound images (18). The training set was composed of 248 ultrasound images from 124 patients while the testing set had 90 ultrasound images from 45 patients (for a total of 338 ultrasound images from 169 primary breast cancer patients) and achieved a sensitivity of 65.5%, a specificity of 78.9%, and an AUC of 0.72 (18).

Other retrospective studies investigating the prediction of lymph node metastasis in breast cancer patients using ultrasound images and AI algorithms to evaluate the images prove more robust in their methodologies through enrolling larger numbers of patients into their training and validation sets. A study by Ashokkumar et al. evaluated three different deep Artificial Neural Networks (ANNs)—one based on feed forward, one on radial basis function, and one on Kohonen self-organizing models—for use in predicting metastasis in pre-operative breast cancer patients and compared their performance against experienced radiologists (19). The study involved a total of 908 images from 750 patients for training the sets. The Kohonen self-organizing model outperformed the others based on the sensitivity of 98%, specificity of 99%, and an AUC of 0.97. For comparison, the feed forward and radial basis function model had a sensitivity of 92% and 90%, specificity of 89% and 95%, and an AUC of 0.95 and 0.94, respectively (19). An additional study by Guo et al. identified 937 breast cancer patients with prior ultrasound images to train and test two different DL radiomics models for evaluating sentinel lymph node and non-sentinel lymph node metastasis, respectively (20). The performance of the DL radiomics model in predicting risk of metastasis with sentinel lymph nodes had a sensitivity of 87.8% and an AUC of 0.86 during training and a sensitivity of 89.7% and an AUC of 0.81 in testing; in non-sentinel lymph nodes the model had a sensitivity of 100% and an AUC of 0.91 during training and a sensitivity of 98.4% and an AUC of 0.81 in testing. This study was notable for its aim to identify the non-sentinel lymph node metastasis *via* DL radiomics obtained from ultrasound images in comparison to previous studies that focus on pathological indicators of sentinel lymph nodes for prediction of the metastatic status of non-sentinel lymph nodes (20). The study by Zhou et al. involves investigating three different CNNs—Inception V3, Inception-ResNet V2, and ResNet-101 architectures—in their performance of predicting negative axillary lymph node metastasis from primary breast cancer ultrasound images (7). The retrospective study involves a total of 1,055 ultrasound images from 834 patients and two different clinical sites. The results were then compared with readings from five experienced radiologists and metastatic status for all patients was confirmed with pathology. The CNN model Inception V3 performed best with an 85% sensitivity, 73% specificity and an AUC of 0.89. For comparison, radiologists performed with an average sensitivity and specificity of 73% and 63%, respectively (7). These larger scale studies indicate that DL models evaluating ultrasound images show promise as an early diagnostic tool when gauging lymph node status in primary breast cancer patients.

AI models can be further enhanced through integrating multiple ultrasound modalities. A study by Zheng et al. combines DL radiomics of conventional ultrasound and shear wave elastography to predict axillary lymph node metastasis pre-operatively. Shear wave elastography provides information about the elasticity of the tissue, in this case the stiffness of the tumor, that it visualizes (6).. A higher shear wave velocity has been correlated with a higher probability of metastasis. This study demonstrated that clinical parameter combined DL radiomics was able to accurately predict the metastatic status of axillary lymph nodes with an AUC of 0.902 and can even differentiate between axillary lymph nodes of low burden of metastasis from those with a high burden of metastasis with an AUC of 0.905 (6). These performance metrics are noticeably higher than either ultrasound alone (AUC of 0.585–0.719) and shear wave elastography alone (AUC of 0.759) in determining axillary lymph node status (6). This indicates that clinical parameter combined DL radiomics on multi-modalities might be the preferred avenue to investigate in future studies for the optimization of a pre-operative clinical diagnosis.

Though ultrasound is the primary imaging tool for examining axillary lymph nodes in breast cancer patients, research extends into DL algorithms for imaging modalities beyond ultrasound. Computed tomography (CT), though it exposes patients to ionizing radiation, has the advantage of acquiring more detail and range than ultrasound while requiring both less time and cost than magnetic resonance imaging (MRI). The study by Liu et al. represents the first attempt to predict axillary lymph node metastasis with a contrast enhanced CT (CECT) through deep learning architecture, and the study compared the results to the performance of traditional machine learning algorithms (21). 401 breast cancer patients were included in the study for a total of 800 axillary lymph node CECT images; the images were divided into 480 training, 160 validation, and 160 test sets. The novel deformable attention VGG19 (DA-VGG19) algorithm used in this study displayed an accuracy of 0.9088, a sensitivity of 0.9500, a specificity of 0.8675, and an AUC of 0.9694, outperforming other traditional models (including Random Forest, DenseNet, ResNet, VGG16, and VGG19, to name a few). In future studies, the authors mention the goal of multi-center studies to further validate their algorithm (21). The study by Yang et al. applied a CNN fast (CNN-F) to determine both the presence and extent of metastasis within sentinel lymph nodes based on CECT (22). The DL model predicting metastasis performed with an AUC 0.817 during validation. Additionally, the study attempted to distinguish metastatic lesions from each other to determine the number of metastatic lesions in sentinel lymph nodes, which had moderate success with an AUC of 0.770 (22). A retrospective study by Li et al. examined breast cancer patients that had undergone 2-deoxy-2-(^18^F)fluoro-D-glucose positron emission tomography/computed tomography [2-(^18^F)FDG-PET/CT] preoperatively and designed a CNN that differentiated between lymph node metastasis and primary breast cancer within the scans (3). The AI model (with an AUC of 0.868) alone did not outcompete the two clinicians, however, when the clinicians worked toward a diagnosis collaboratively with the AI model their sensitivities improved from 59.8% and 57.4% to 68.6% and 64.2%, respectively (3). Clinician specificity remained at 99.0% and 99.5%, respectively regardless of working with the AI model (3). This study demonstrates how the endpoint in developing AI models is not to replace clinicians as the primary diagnostician, but to instead enhance clinicians' performance through AI-assisted diagnoses.

MRI is another imaging modality that has been used in the diagnosis of axillary lymph node metastasis for breast cancer patients, however, unlike the other modalities discussed previously, MRI has low inter-observer variability, no ionizing radiation, and improved diagnostic contrast (13). The study by Ren et al. develops a CNN approach to identify axillary lymph node metastasis on breast MRI images with a specificity of 79.3% ± 5.1%, a sensitivity of 92.1% ± 2.9%, and an AUC of 0.91 ± 0.02 (23). They compare these metrics to those of an experienced radiologist and radiologist resident reading the same MRI images: a specificity of 75% and 54%, respectively, a sensitivity of 81% and 78%, respectively, and an AUC of 0.80 and 0.73, respectively (23). The CNN outperformed both physicians within this study. Yu et al. examines radiomic signatures in dynamic contrast-enhanced MRI (DCE-MRI) that can detect axillary lymph node metastasis in early breast cancer patients within their study (24). This study operated on a larger scale in comparison to the other articles discussed throughout the review, as it involved 1,214 patients from 4 different medical facilities, thus increasing the statistical power. The authors combined radiomic and clinical signatures to create a novel clinical-radiomic nomogram that identified axillary lymph node metastasis with an AUC of 0.90 during validation, as opposed to using radiomic signatures or clinical signatures alone which performed with an AUC of 0.85 and 0.71, respectively (24). Furthermore, this study also examined disease free survival, and developed a clinical-radiomic nomogram that predicted disease free survival 3 years following a breast cancer diagnosis that performed with an AUC of 0.90 during validation (24). Similarly, a study by Song et al. combined radiomics features from DCE-MRI with various clinical factors to identify metastasis in axillary lymph nodes (25). The dataset was smaller than the previous mentioned study, with a total of 432 patients, 296 used for training and 136 used for testing, and the combined clinical model and radiomic signature created a multivariable model that performed with an AUC of 0.874 (25). The success of these studies illustrates the power of multi-omics analysis in predicting both the presence and the course of a disease.

The included table (Table 1) gives a summary of the recent literature presented throughout this review.

**Table 1:** Summary table of recent literature discussed in review comparing different AI methods and AUC for identifying axillary lymph node metastasis.

Paper	Imaging Modality	Number of Patients	AI Methods	AUC
Ashokkumar et al.	Ultrasound	750	Kohonen self-organizing model	0.97
Guo et al.	Ultrasound	937	Pretrained ImageNet and Deep Learning Radiomics	0.81
Sun et al.	Ultrasound	169	CNN with Adam Optimizer training	0.72
Tahmasebi et al.	Ultrasound	296	Google AutoML vision platform	Not given
Zheng et al.	Ultrasound	584	Pretrained ResNet combined with clinical features	0.902
Zhou et al.	Ultrasound	834	Inception V3 CNN	0.89
Ren et al.	MRI	99	Standard 2D CNN	0.91 ± 0.02
Song et al.	DCE-MRI	432	Radiomics feature selection using LASSO algorithm	0.874
Yu et al.	DCE-MRI	1,214	Radiomics-based survival analysis	0.90
Li et al.	FDG-PET/CT	407	3D residual CNN with attention module	0.868
Liu et al.	CECT	401	Deformable attention VGG19	0.9694
Yang et al.	CECT	348	Pretrained AlexNet with logistic regression	0.817

**Figure 1:**
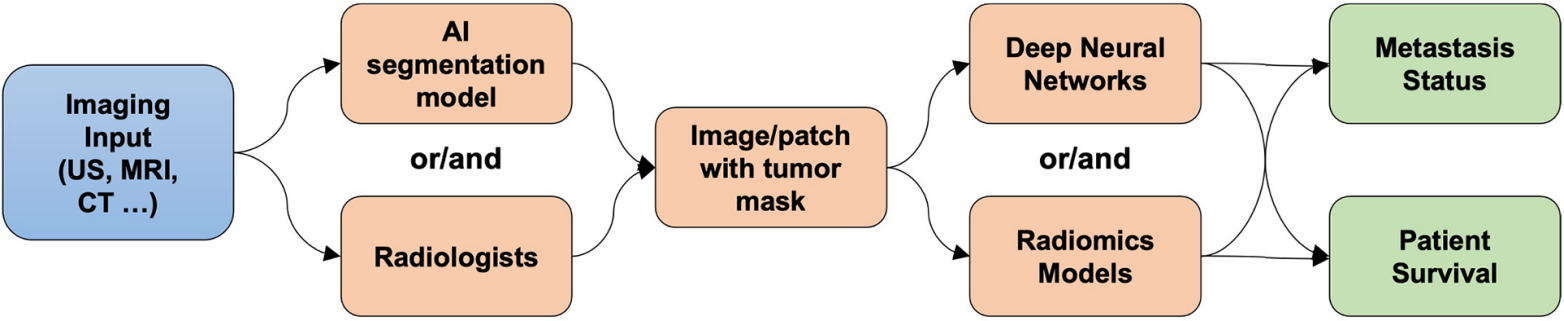
Example workflow illustrating AI analysis in identifying axillary lymph node metastasis.

The included figure (Figure 1) illustrates an example of the workflow in AI analysis in identifying axillary lymph node metastasis.

## Potential future developments

Many of the articles presented throughout this review were retrospective studies, leading to heterogeneity within different images of the same modality within a given study. Not everything was completely standardized, and this likely presented confounding variables within the studies. A potential future project would work to eliminate the variability. Moreover, the datasets used to train AI models should exhibit more inclusivity. The patient population with breast cancer includes people of varying backgrounds, which often means significant differences in characteristics like types of breast cancer, breast density, and age groups, to name a few. Future studies would expand the dataset to include a larger and more diverse patient population and multiple sites to increase the generalizability and the power of the study.

The studies examined that found the greatest success in their purpose tended to involve multiple imagining modalities, multi-omics analyses, or significant collaboration between clinician and AI model in the work up to a diagnosis, indicating that involving multiple perspectives or viewpoints leads to a superior performance. Future developments in AI models that predict axillary lymph node metastatic status should work to incorporate multiple modalities or signatures for a better predictive power.

## Conclusion

The growing popularity of AI in the medical imaging field is driving innovation toward creating DL models that can predict the metastasis of breast cancer to the axillary lymph nodes preoperatively, reducing the number of patients that undergo unnecessary and invasive procedures. Some preliminary models demonstrate early success, achieving a greater sensitivity and specificity when compared to experienced radiologists. However, more work is necessary to repeat such studies on larger scales with more standardized variables and interpretability before they can be clinically useful. Besides, considering the heterogeneity, e.g., vendors, demographic information, and scanning protocols, multi-cohort studies with data harmonization strategies will further improve the generalization of AI solutions for deployment in clinical settings.
